# Cultural impacts on shared decision-making: A cross-European study of psychiatrist preferences in 38 countries

**DOI:** 10.1192/j.eurpsy.2025.10082

**Published:** 2025-08-11

**Authors:** Yasuhiro Kotera, Christopher Newby, Martina Rojnic Kuzman, Philip Gorwood, Andrea Fiorillo, Mike Slade

**Affiliations:** 1 School of Health Sciences, University of Nottingham, Nottingham, UK; 2 Center for Infectious Disease Education and Research, Osaka University, Suita, Japan; 3 Department of Social Sciences, Azerbaijan University, Baku, Azerbaijan; 4 School of Medicine, University of Nottingham, Queen’s Medical Centre, Nottingham, UK; 5 Department of Psychiatry, Zagreb University Hospital Centre, Zagreb, Croatia; 6 Institute of Psychiatry and Neuroscience of Paris (IPNP), Université Paris Cité, Paris, France; 7 GHU-Paris Psychiatrie et Neurosciences, Hôpital Sainte Anne, Paris, France; 8 Department of Psychiatry, University of Campania “L. Vanvitelli”, Napoli, Italy; 9 Faculty of Nursing and Health Sciences, Health and Community Participation Division, Nord University, Namsos, Norway

**Keywords:** cross-cultural psychiatry, individualism, indulgent culture, low power distance, shared decision-making

## Abstract

**Background:**

Shared decision-making (SDM) is a collaborative process between clinicians and service users to select treatment, guided by evidence and service user preferences. SDM has clinical, economic, and ethical benefits compared to clinician-led decision-making; yet, implementation remains challenging. An important knowledge gap is the influence of culture on implementation. Cultural impacts on service user decision-making preferences have been documented, but little is known about how culture impacts clinician preferences. This study examined associations between country-level cultural characteristics and decision-making preferences of psychiatrists in routine care settings across Europe.

**Methods:**

We analysed data from 751 psychiatrists and trainees in 38 European countries, who completed the Clinical Decision-Making Style–Staff (CDMS-S) scale. Country-level Hofstede cultural dimensions were linked to CDMS-S scores using univariate and multivariate regression models. Mixed-effects models were used to account for country-level clustering and controlling for professional and economic variables.

**Results:**

In univariate analyses, all six dimensions were associated with SDM preferences. However, only three remained significant in mixed-effects models. Higher levels of Indulgence and Individualism were associated with stronger preferences for SDM, while higher Power Distance was associated with more clinician-led decision-making. These associations did not remain significant in fully adjusted multivariate models, suggesting professional and systematic factors mediate cultural influences.

**Conclusions:**

Indulgence, Individualism, and Power Distance are associated with psychiatrists’ decision-making preferences across Europe. Culturally sensitive SDM interventions should address not only clinician attitudes but also healthcare structures and patient expectations. Findings offer an empirical foundation for tailoring SDM training and policy to diverse cultural contexts within European psychiatry.

## Introduction

Clinical shared decision-making (SDM) occurs in most encounters between clinicians and service users. Involvement can range from clinician-led decision-making, through SDM in which decisions are jointly made, to active participation in which the service user leads the decision-making process. SDM is “a process in which clinicians and patients work together to select tests, treatments, management or support packages, based on clinical evidence and the patient’s informed preferences” [1]. Clinicians in this context include different professional groups of clinicians, including psychiatrists. SDM preferences reflect the desired level of active service user participation in the decision-making process, which can vary significantly across psychiatrists and cultures.^2^ Patients, more commonly now described as service users, include their informal carers [2]. SDM empowers service users to engage with the care they receive, helping them make informed choices aligned with their values and preferences. The human rights of service users can be more protected in SDM, and increasingly, service users prefer SDM rather than clinicians making decisions for them [2]. SDM involves service users as experts in their own experiences, requiring their active involvement in treatment [1]. Higher service user involvement is associated with multiple positive outcomes, including improved efficiency and quality of health services, enhanced quality of life and satisfaction in service users, and reduced hospital admissions [3, 4]. SDM is advantageous clinically, economically, and ethically.

SDM research is increasing: 229 publications in 2009 compared with 1,199 in 2018 [5]. SDM research in mental health has been growing at a similar pace: 4 publications in 2003 compared with 33 in 2015 [6]. A systematic review of 14 randomised controlled trials for service users with mood disorders identified that all SDM interventions improved depression or medication adherence [7]. A systematic review on SDM for adolescent mental healthcare reported positive effects, including treatment satisfaction, better mental health understanding, and reduced depression symptoms [8]. A meta-analysis for digital interventions supporting SDM found a significant effect on patient engagement, symptom reduction, and decision process [9]. Weaknesses of SDM research have also been identified, including poor study quality [10] arising in part from poor implementation of SDM interventions [11].

One reason for poor implementation of SDM is an underdeveloped understanding of cultural impacts [12]. Culture is understood as “*the sum of attitudes, customs and beliefs that distinguishes one group of people from another*” [13]. There are cultural challenges in SDM: SDM is derived from highly individualistic cultural values; therefore, it can cause harm if used by or for people oriented to other cultural values [14]. What is considered important in SDM varies significantly across cultures. As an example, service users from a White background often value the belief that doing everything possible is crucial, while those from an Asian background tend to prioritise avoiding worry in SDM [13]. Service users in minority cultural groups often experience systemic barriers that reduce trust in healthcare systems, which can impact communication with healthcare professionals and impede SDM [15]. Moreover, SDM expectations can vary by culture. Service users in some minority cultural groups may feel more comfortable with a less active role in SDM compared to those in the dominant cultural group [16]. Expectations about other factors, such as involvement of family members, comfortable speed of decision-making, degrees of loss aversion (sensitivity to possible loss), and spread of the alternatives (justifying their decision by perceiving their own choice positively and the other unchosen choices negatively), can also differ by cultures [17].

The impact of culture on psychiatrist SDM preferences is under-researched. A systematic review identified 24 components of SDM; however, almost all components focus on understanding service users, such as “Patient preferences,” “Tailor information,” and “Learn about the patient” [18]. Only one component, “Healthcare professional preferences,” is related to psychiatrists [18]. To understand and address cultural impacts on SDM implementation, it is essential to have a better understanding of how culture impacts psychiatrists’ SDM style [19]. Our previous study found regional variations in psychiatrists’ preferences for SDM across Europe [20]. Psychiatrists in Northern and Western Europe showed stronger preferences for active service user participation in SDM compared to their counterparts in other European regions. However, beyond these broad regional categorisations, the impact of culture on psychiatrist SDM preferences remains unexplored.

The importance of understanding cultural impact on psychiatrist SDM preferences has been increasingly recognised. In an SDM process, psychiatrists collaboratively communicate with service users and other staff. Psychiatrists today work with staff and service users from different cultural backgrounds more frequently than before, facilitated by the increasing migrant populations and integration of social and health services, both of which are salient in Europe [21]. A lack of shared approaches to mental illness among staff and service users from diverse cultural backgrounds can impede SDM [22]. Additionally, more and more psychiatry trainees are considering moving to another country. Two-thirds of psychiatrist trainees in Europe (*n* = 2,281 from 33 countries) have ever considered leaving the country in which they currently live, and over a quarter of them have taken practical steps towards leaving [23]. These findings indicate an increasing need for understanding cultural impact on psychiatrists’ SDM preferences.

Hofstede’s cultural dimension theory is the most established quantitative framework in cross-cultural research, with more than 46,000 citations [24]. The cultural dimension theory proposes six dimensions (Table 1).

**Table 1.** Six dimensions in the cultural dimension theory

Dimension (interpretation)	Meaning
Indulgence (vs. restraint)	Acceptance of relatively free gratification of basic and natural human desires to enjoy life.
Long-term orientation (vs. short-term orientation)	Values oriented towards future rewards, perseverance, and thrift, which are related to “saving” as opposed to “spending.”
Uncertainty avoidance (high vs. low)	The degree to which individuals feel threatened by unknown situations, and try to avoid such situations.
Success drivenness (vs. quality orientation)	A societal value for achievement and material rewards for success (originally named “Masculinity”).
Individualism (vs. collectivism)	The degree to which a society expects individuals to be loosely tied to one another, and to take care of only themselves and their immediate family.
Power distance (high vs. low)	The degree to which inequality and unequal distributions of power between parties are accepted.

Cultural impacts become even more important given the trend towards global mobility. The setting of this study was in Europe, which is a multi-cultural region: as of 1 January 2021, 24 million people from non-European Union (EU) countries were living in one of the EU countries, accounting for 5.3% of the EU population. Additionally, 14 million people were living in one of the EU countries with the citizenship of another EU country [25]. These figures are on an increasing trend, for example, an 18% increase for non-EU citizens moving to an EU country, and a 17% increase for EU citizens moving to another EU country, comparing 2020 with 2021 [25]. Non-EU countries, such as the United Kingdom and Switzerland, also have high rates of residents who were born outside the countries (UK 14% and Switzerland 30%) [26]. Moreover, the quality and process of psychiatric practice, and the socio-economic status are relatively similar across countries [27]. These aspects support this cross-cultural research to be conducted in this area.

### Study aim

This study aimed to examine the association between Hofstede’s six cultural dimensions and psychiatrists’ preferences for active service user participation in SDM across Europe.

## Methods

A cross-sectional study using the data collected in the European Psychiatric Association (EPA) Ambassadors Survey and Hofstede’s metrics. The EPA data were reported to show the preference for SDM among psychiatrists in the three regions [20]. The Strengthening the Reporting of Observational Studies in Epidemiology (STROBE) guidelines were followed (Supplementary Material 1).

### Procedures

The EPA invited psychiatrists across Europe who were part of the EPA community to participate, including individual EPA members, members of its affiliated associations, and the ~5,000 attendees of the last 10 EPA congresses. In 2020, this group was invited to become EPA Ambassadors and to participate in EPA surveys. An invitation email was sent to EPA Ambassadors, the Council of National Psychiatric Associations, the EPA Board, and the EPA Sections, with a request to further distribute the invitation among their associated members. Responses were gathered from April to December 2021 via an online questionnaire. The study was open to all mental health professionals (psychiatrists, psychiatry trainees, psychologists, social workers, and nurses) working in Europe. Only responses from psychiatrists were included in the current study. The authors confirm that all procedures followed in this research align with the ethical standards of the relevant national and institutional committees on human experimentation, as well as the 1975 Helsinki Declaration, revised in 2008 and 2013. The study was approved by the Ethical Committee of the Zagreb University Hospital Centre (Ref. 02/013-JG).

### Measures

The *outcome variable* was SDM preference, assessed using the Clinical Decision-Making Style–Staff (CDMS-S) questionnaire [28], which measures “Participation in decision-making” through two subscales (Sections A and B), with all items rated on 5-point Likert scales. Section A consists of six items (rated from “*strongly disagree*” to “*strongly agree*”) that reflect general preferences for decision-making in routine mental health services. Section B includes nine items (rated from “*service user*” to “*me*”) that measure specific preferences for decision-making based on three clinical vignettes (focusing on work, medication side effects, and general medication use). Items 1, 2, 3, and 5 are reverse-scored. The “Participation in decision-making” subscale is calculated as the prorated mean of all items in Sections A and B, provided at least 12 out of the 15 items have been completed. The score ranges from 0 to 4, with higher scores indicating a greater preference by the clinician for active involvement of service users in decision-making.

The *predictor variables* were collected in two sets. The first set was cultural dimensions, obtained from Hofstede (https://geerthofstede.com/research-and-vsm/vsm-2013/). Hofstede’s dataset provides scores for the six cultural dimensions of 111 countries. The data were collected using the Value Survey Module 2013, a 24-item self-report measure, responded on a 5-point Likert scale from 1 to 5 [29]. Each dimension score is calculated using index formulas in the manual. The scores are presented from 0 (low compared to other countries) to 100 [29]. Data for all 38 countries are appended (Supplementary Material 2). The second set was collected in the EPA survey, comprising socio-demographics (age, sex, and city size) and professional characteristics: expertise, training, and practice (time since becoming consultant psychiatrists, subspecialty, job position, type of practice, clinical setting, and duration of appointments).

Two *confounder variables* were included in the fully adjusted analyses, chosen for their relevance to Hofstede’s metrics and mental health care resources [30]. The percentage of gross domestic product (GDP) spent on health represents the proportion of a country’s economy allocated to healthcare, calculated by dividing total health expenditure by GDP (https://data.worldbank.org/indicator/SH.XPD.CHEX.GD.ZS). The Gini coefficient, sourced from the World Bank (https://data.worldbank.org/indicator/SI.POV.GINI), measures income inequality within a country, ranging from 0 (*indicating perfect equality*) to 1 (*representing maximum inequality*).

### Statistical methodology

The survey data [20] was linked by country with the country-specific Hofstede metrics, and all analyses were carried out using the R language package. The outcome variable used was the CDMS-S score.

First, we performed univariate complete-case weighted regression analyses (Model 1), applying the same country weights as used in our previous study [20]. In parallel, we ran a univariate mixed-effects regression model (Model 2) to account for clustering of cultural dimensions within countries, again using the complete-case dataset.

Second, we conducted multivariate analyses for both Models 1 and 2, incorporating the cultural dimensions and EPA variables from the final model in Kuzman et al. [20]. These variables included European region, sex, age, city size, profession type, practice type, patient type, specialised condition, specific specialist conditions, provision of psychotherapy, time since obtaining specialist psychiatric qualification, and frequency of appointments. In addition, we included two confounding variables: the Gini coefficient and GDP expenditure on health.

Third, we addressed missing data through multiple imputation using the MICE package in R. Cultural dimensions, EPA variables, and the two confounders (Gini coefficient and GDP on health) were used as predictors in the imputation model. We generated 50 imputed datasets and applied both univariate and multivariate versions of Models 1 and 2 to each. Final estimates were pooled using Rubin’s rules.

We report *β*-coefficients, along with 95% confidence intervals (CIs) and corresponding *p*-values.

## Results

### Participant information

The online survey was completed by 919 participants across 38 European countries. After excluding 27 (3.0%) psychologists, 14 (1.5%) other mental health professionals, and 10 (1.1%) participants with unspecified professions, the final sample comprised 738 (81.2%) psychiatrists and 130 (14.3%) psychiatry trainees, totalling 868 participants from the target population. Complete data on all 15 CDMS-S items were missing for 112 out of 868 (12.9%) respondents. Additionally, five participants with incomplete data on 14 CDMS-S items (*n* = 2), 13 items (*n* = 1), and 10 items (*n* = 2) were excluded. This resulted in a final sample of 751 (86.5%) out of 868 participants for analysis. In the final sample, 322 (42.9%) participants were from Central and Eastern Europe, 273 (36.4%) from Northern and Western Europe, and 156 (20.8%) from Southern Europe (Table 2; the countries categorised in each region are listed in Supplementary Material 2).

**Table 2.** Participant characteristics

Category	Subcategory	Whole sample (*n* = 751)	Central and Eastern (*n* = 322)	Northern and Western (*n* = 273)	Southern (*n* = 156)
Sex	Women	357 (47.8%)	162 (50.5%)	118 (43.5%)	77 (49.7%)
	Men	390 (52.2%)	159 (49.5%)	153 (56.5%)	78 (50.3%)
Age	18–29	80 (10.7%)	38 (11.8%)	18 (6.6%)	24 (15.4%)
	30–39	222 (29.6%)	96 (29.8%)	74 (27.1%)	52 (33.3%)
	40–49	181 (24.1%)	79 (24.5%)	69 (25.4%)	33 (21.2%)
	50–59	167 (22.3%)	73 (22.7%)	68 (25.0%)	26 (16.7%)
	60+	100 (13.3%)	36 (11.2%)	43 (15.8%)	21 (13.5%)
City size	≤100,000	164 (21.9%)	65 (20.2%)	85 (31.3%)	14 (9.0%)
	>100,000	584 (78.1%)	256 (79.8%)	187 (68.7%)	141 (91.0%)
Profession	Psychiatrist	643 (85.6%)	275 (85.4%)	243 (89.0%)	125 (80.1%)
	Psychiatry specialist trainee	108 (14.4%)	47 (14.6%)	30 (11.0%)	31 (19.9%)
Practice	Public	466 (62.3%)	177 (55.1%)	190 (69.9%)	99 (63.5%)
	Mixed	146 (19.5%)	77 (24.0%)	31 (11.4%)	38 (24.4%)
	Private	99 (13.2%)	54 (16.8%)	29 (10.7%)	36 (12.1%)
	Community setting	37 (4.9%)	13 (4.0%)	22 (8.1%)	2 (1.3%)
Patients	Mainly outpatients	469 (62.8%)	189 (58.9%)	163 (60.1%)	117 (75.5%)
	Mainly inpatients	278 (37.2%)	132 (41.1%)	108 (39.9%)	38 (24.5%)
Subspecialty	Specialized	485 (64.7%)	199 (61.8%)	188 (68.9%)	99 (63.2%)
	Mood and anxiety disorders	152 (20.3%)	74 (23.0%)	56 (20.5%)	22 (14.2%)
	Psychosis	126 (16.8)	57 (17.7%)	46 (16.8%)	23 (14.8%)
	Child psychiatry	68 (9.1%)	33 (10.2%)	15 (5.5%)	20 (12.9%)
	Addiction	47 (6.3%)	18 (5.6%)	20 (7.3%)	9 (5.8%)
	Other	92 (12.3%)	17 (5.3%)	51 (18.7%)	24 (15.5%)
	Unspecialized	265 (35.3%)	123 (38.2%)	85 (31.1%)	57 (36.8%)
Providing psychotherapy		315 (42.0%)	124 (38.5%)	120 (44.0%)	71 (45.8%)
Time since specialist psychiatric qualification (years), median (IQR)		13 (5; 25)	15 (5; 25)	14 (6; 26)	10 (4; 24)
Average frequency of clinical appointments with each patient, *n* (%)	Several times a week	113 (15.3%)	66 (20.8%)	28 (10.5%)	19 (12.3%)
	Several times a month	195 (26.4%)	84 (26.5%)	85 (31.1%)	26 (16.8%)
	Once a month	308 (41.8%)	144 (45.4%)	98 (36.7%)	67 (43.2%)
	Less frequent	122 (16.5%)	23 (7.3%)	56 (21.0%)	43 (27.7%)
Duration of visit (min), median (IQR)		35 (30–45)	31 (30–45)	40 (30–50)	30 (20–45)

Abbreviation: IQR, interquartile range.

*Note*: Data in this table have been published in Table 1 in Kuzman et al. [20].

*Note*: Data were missing for sex in 4 (0.5%), age in 1 (0.1%), city size in 3 (0.4%), practice in 3 (0.4%), patients in 4 (0.5%), subspecialty in 1 (0.1%), time since specialist psychiatry qualification in 10 (1.3%), cost of visit in 271 (36.1%), and average frequency of clinical appointments with each patient in 12 (1.6%) participants.

### Culture and SDM

Univariate analysis results for complete case analysis using Models 1 and 2, and multiple imputation estimates for Model 1, can be found in Table 3. It was not possible to pool the multiple imputed datasets for Model 2 for univariate or multivariate results due to the clustered nature of the data. All of the cultural dimension variables were significant in the univariate weighted linear regression (Model 1) for both complete and multiple imputed datasets. However, when adjusted for the EPA variables and covariates (Gini coefficient and GDP on health), none of the cultural dimension variables were significant, suggesting that the more specific variables within each country predicted the CDMS-S score better than the more general country cultural dimensions, which is to be expected.

**Table 3.** Predictors of SDM

	Univariate, complete case, weighted linear regression (Model 1)		Univariate, multiple imputation, weighted linear regression (Model 1)		Univariate, complete case, linear missed effect (Model 2)	
Cultural dimensions	*β* (95% CI lower, upper)	*p*	*β* (95% CI lower, upper)	*p*	*β* (95% CI lower, upper)	*p*
Indulgence	**0.009 (0.006, 0.012)**	**<0.001**	**0.009 (0.006, 0.012)**	**<0.001**	**0.005 (0.002, 0.009)**	**0.005**
Long-term orientation	**0.006 (0.003, 0.008)**	**0.005**	**0.005 (0.003, 0.008)**	**<0.001**	0.00001 (−0.004, 0.004)	0.997
Uncertainty avoidance	**−0.009 (−0.011, 0.006)**	**<0.001**	**−0.008 (−0.011, −0.006)**	**<0.001**	−0.004 (−0.007, 0.0001)	0.049
Success drivenness	**0.005 (0.002, 0.007)**	**<0.001**	**0.004 (0.001, 0.006)**	**0.002**	−0.001 (−0.004, 0.002)	0.050
Individualism	**0.008 (0.005, 0.010)**	**<0.001**	**0.007 (0.005, 0.010)**	**<0.001**	**0.007 (0.003, 0.011)**	**0.003**
Power distance	**−0.009 (−0.011, −0.007)**	**<0.001**	**−0.008 (−0.010, −0.006)**	**<0.001**	**−0.005 (−0.008, −0.003)**	**0.001**

*Note:* Significant values (*p* < 0.05) are bold. *Β*, *β*-coefficient; CI, confidence interval.

The weighted linear regression model, however, did not capture the within- and between-country cluster variation. By using a univariate mixed-effect model (Model 2), we adjusted for country as a random effect. In this model for the complete case dataset, we found that the cultural dimension variables Indulgence (*β* = 0.005, 95% CI = 0.002 to 0.009, *p* = 0.005) and Individualism (*β* = 0.007, 95% CI = 0.003 to 0.011, *p* = 0.003) were significantly positively associated with CDMS-S score. Power Distance (*β* = −0.005, CI = −0.008 to −0.003, *p* = 0.001) was significantly negatively associated with CDMS-S scores. Although statistically significant, the effect sizes were modest, with standardised *β*-coefficients of 0.19, 0.24, and 0.25 for Indulgence, Individualism, and Power Distance, respectively. All cultural dimension variables were not significant after adjusting for the EPA variables and confounding variables in a multivariate complete case mixed-effect model.

In sum, all six cultural dimensions were associated with SDM in univariate models. However, these associations disappeared in multivariate models that included professional and system-level variables (EPA, Gini, and GDP on health). Mixed-effects modelling, which accounted for country-level clustering, showed that higher Indulgence and Individualism and lower Power Distance were linked to greater SDM.

## Discussion

This cross-cultural study investigated the association between national cultural dimensions and shared SDM among psychiatrists and psychiatry trainees across 38 European countries. Using Hofstede’s cultural framework, we examined how six cultural dimensions are related to clinicians’ preference for active involvement from patients in SDM, as measured by CDMS-S. Our analyses revealed significant associations in univariate models, with all six dimensions showing a relationship with SDM scores. However, these associations did not persist in multivariate models that included clinician-level variables and health system indicators. Notably, in mixed-effects modelling accounting for country-level clustering, Indulgence and Individualism were positively associated with SDM, while Power Distance showed a negative association.

The finding that all six cultural dimensions were associated with SDM in univariate models suggests that broad national cultural characteristics play a role in shaping clinicians’ general orientation toward patient involvement. This aligns with prior literature suggesting that macro-cultural values influence health professionals’ communication styles, attitudes towards hierarchy, and views on patient autonomy [30, 31]. For instance, in cultures with high Individualism, autonomy and personal agency are more strongly emphasised [32], which may encourage clinicians to pay close attention to each individual’s needs in clinical decision-making. Conversely, high Power Distance cultures may reinforce hierarchical relationships [33] that discourages shared approaches to care.

However, the disappearance of these associations in multivariate models implies that cultural values alone are insufficient to explain SDM practices once more immediate contextual variables, such as professional characteristics (EPA) and economic indicators (Gini and GDP on health), are accounted for. This highlights the importance of distinguishing between distal cultural norms (macro-level influences) and proximal systemic or organisational factors (micro-level influences) [34]. The findings suggest that while national culture sets a backdrop for attitudes and expectations, the actual implementation of SDM may also need to consider professional education, institutional policies, and healthcare infrastructure.

The mixed-effects model further refined our understanding by accounting for country-level clustering. Here, three cultural dimensions remained significant: Indulgence, Individualism, and Power Distance. These findings are consistent with expectations and previous work.

Indulgence, which reflects a cultural orientation towards enjoyment, freedom, and self-expression, was also associated with stronger SDM preferences. Conversely, its opposite, Restraint, was linked to lower preferences for SDM. In Restraint cultures, social norms emphasise self-discipline and the suppression of gratification, which may lead to more conservative, clinician-led approaches to decision-making and limit the perceived legitimacy of patient preferences in clinical interactions [35]. Countries higher in Indulgence may promote clinical environments where patients’ subjective experiences and personal values are welcomed and legitimised [36]. This orientation could foster more open, empathetic, and participatory consultation styles, aligning well with the principles of SDM.

High Individualism was associated with greater SDM, suggesting that clinicians in countries where autonomy and self-determination are emphasised are more likely to involve patients in decision-making processes. The most Individualistic countries in our sample, based on Hofstede, were the United Kingdom, Hungary, and the Netherlands. This finding aligns with previous research that associates individualistic values with patient-centred communication and greater emphasis on equality in professional relationships [37]. In contrast, countries with lower levels of Individualism, such as Serbia, Portugal, and Slovenia, may place more value on relational or collective processes, which can also support SDM but in different culturally situated ways. These patterns highlight the need to interpret SDM preferences through the lens of local cultural norms, rather than assuming a universal model tied exclusively to Western ideals.

Higher Power Distance was associated with more clinician-led decision-making. Slovakia, Serbia, and Russia were the highest Power Distance participating countries, while Austria, Denmark, and Ireland were the lowest. High Power Distance countries are characterised by hierarchical structures where authority figures, including psychiatrists, are expected to lead and direct decisions [38]. In these contexts, SDM might be seen as challenging established norms of authority and trust in the expertise of clinicians. Conversely, psychiatrists working in low Power Distance cultures (e.g., Austria) may perceive SDM as aligning with broader societal values of democracy, fairness, and patient autonomy [39]. These findings underscore the nuanced ways cultural dimensions shape professional attitudes and clinical practices.

Additionally, SDM in high Power Distance settings may be further influenced by patient expectations. In hierarchical cultures, patients might prefer a more directive approach, reinforcing the clinician-led decision-making norm [40]. This contrasts with low Power Distance contexts, where patients may actively expect to collaborate and share responsibility in decision-making. Such differences highlight how both clinician and patient perspectives, shaped by cultural norms, contribute to the enactment of SDM. This adds depth to our previous study findings [20] based on geographical categories, demonstrating the value of incorporating cultural dimensions like Power Distance into models of decision-making in psychiatry. Together, these observations highlight how Power Distance impacts both psychiatrists’ decision-making preferences and the cultural alignment of SDM with societal expectations.

The lack of significance in the multivariate mixed-effects model again reinforces the notion that cultural values may exert an indirect rather than direct influence on clinical practice. This is in line with theoretical models that position culture as a distal factor, operating through more immediate social and structural mechanisms [41]. Our findings also suggest that interventions aimed at enhancing SDM should not rely solely on cultural awareness but must also address local structural conditions, including training, workload, and institutional norms.

From a policy perspective, this study provides useful insights for international efforts to promote SDM in psychiatry. While cultural competence remains important, it must be complemented by context-sensitive strategies that recognise the influence of systemic variables [42]. For instance, countries with high Power Distance might benefit from institutional reforms that empower patients and restructure clinician–patient hierarchies, alongside cultural education. Similarly, in countries with lower Indulgence scores, promoting patient-centredness may require efforts to legitimise the expression of personal preferences in clinical encounters. These results also have implications for training and education. Our findings support the idea that while cultural context influences attitudes towards SDM, these attitudes are modifiable and interact with professional development. Embedding SDM principles into psychiatric training across Europe, tailored to local cultural and systemic contexts, may help bridge the gap between global best practices and local realities [43].

Several limitations should be noted. First, while Hofstede’s framework provides a useful heuristic, it may oversimplify the complexity of national cultures and does not account for intracultural variation. Second, the cross-sectional design limits causal inference. Third, while we included both psychiatrists and trainees, responses were self-reported and may reflect social desirability bias. Fourth, although our models included economic indicators, other factors such as legal frameworks or specific national mental health policies were not captured. Lastly, we were unable to pool imputed data for mixed-effects models due to technical constraints, which limited the robustness of those estimates. Despite these limitations, this study makes a novel and timely contribution to understanding how cultural and systemic factors jointly shape SDM in psychiatry. It represents one of the first large-scale, empirical investigations to apply a validated cross-cultural framework to SDM practices in mental health across Europe. The combination of national cultural indicators with clinician-level and structural variables provides a unique, multilevel perspective that bridges gaps between global cultural theory and real-world clinical behaviour. These findings reinforce the importance of contextualising SDM promotion efforts not only within professional education and policy reform but also within the socio-cultural realities of different regions.

In conclusion, while national cultural dimensions are associated with SDM attitudes among psychiatrists in Europe, their influence appears to be mediated or moderated by professional and systemic factors. Mixed-effects analysis identified three key cultural dimensions – Individualism, Indulgence, and Power Distance – as significant predictors, but these associations weakened when adjusting for clinical attitudes and economic conditions. Efforts to promote SDM should, therefore, adopt a multilevel approach that incorporates cultural sensitivity alongside institutional and educational reform. Future research should explore these interactions further, ideally through longitudinal and mixed-method designs that can unpack the pathways linking culture, structure, and clinical behaviour.

## Supporting information

10.1192/j.eurpsy.2025.10082.sm001 Kotera et al. supplementary material Kotera et al. supplementary material

## Data Availability

The data that support the findings of this study are available from the corresponding author upon reasonable request.

## Supplementary material

The supplementary material for this article can be found at http://doi.org/10.1192/j.eurpsy.2025.10082.
