# Supplementary material for: Cultural impacts on shared decision-making: A cross-European study of psychiatrist preferences in 38 countries
Source: Eur Psychiatry. 2025 Aug 11;68(1):e108. doi: 10.1192/j.eurpsy.2025.10082 (PMC12438978; doi:10.1192/j.eurpsy.2025.10082)
Supplement: Kotera et al. supplementary material [file S0924933825100825sup001.docx]

**SUPPLEMENTARY MATERIALS**

**Supplementary Material 1. STROBE checklist**

|  | **Item No.** | **Recommendation** | **Page No.** |
| --- | --- | --- | --- |
| **Title and abstract** | 1 | (a) Indicate the study’s design with a commonly used term in the title or the abstract | 1-2 |
|  |  | (b) Provide in the abstract an informative and balanced summary of what was done and what was found | 2 |
| **Introduction** | | | |
| Background/rationale | 2 | Explain the scientific background and rationale for the investigation being reported | 3-4 |
| Objectives | 3 | State specific objectives, including any prespecified hypotheses | 4 |
| **Methods** | | | |
| Study design | 4 | Present key elements of study design early in the paper | 4 |
| Setting | 5 | Describe the setting, locations, and relevant dates, including periods of recruitment, exposure, follow-up, and data collection | 4-5 |
| Participants | 6 | (a) Cross-sectional study—Give the eligibility criteria, and the sources and methods of selection of participants | 4-5 |
| Variables | 7 | Clearly define all outcomes, exposures, predictors, potential confounders, and effect modifiers. Give diagnostic criteria, if applicable | 5 |
| Data sources/ measurement | 8 | For each variable of interest, give sources of data and details of methods of assessment (measurement). Describe comparability of assessment methods if there is more than one group | 5 |
| Bias | 9 | Describe any efforts to address potential sources of bias | 5-6 |
| Study size | 10 | Explain how the study size was arrived at | 6 |
| Quantitative variables | 11 | Explain how quantitative variables were handled in the analyses. If applicable, describe which groupings were chosen and why | 5 |
| Statistical methods | 12 | (a) Describe all statistical methods, including those used to control for confounding | 5-6 |
|  |  | (b) Describe any methods used to examine subgroups and interactions | N/A |
|  |  | (c) Explain how missing data were addressed | 6 |
|  |  | (d) Cross-sectional study—If applicable, describe analytical methods taking account of sampling strategy | N/A |
|  |  | (e) Describe any sensitivity analyses | N/A |
| Participants | 13 | (a) Report numbers of individuals at each stage of study—e.g. numbers potentially eligible, examined for eligibility, confirmed eligible, included in the study, completing follow-up, and analysed | 6 |
|  |  | (b) Give reasons for non-participation at each stage | NA |
|  |  | (c) Consider use of a flow diagram | N/A |
| Descriptive data | 14 | (a) Give characteristics of study participants (ego demographic, clinical, social) and information on exposures and potential confounders | Table 2 |
|  |  | (b) Indicate number of participants with missing data for each variable of interest | 6 |
|  |  | (c) Cohort study—Summarise follow-up time (e.g., average and total amount) | N/A |
| Outcome data | 15 | *Cross-sectional study—Report numbers of outcome events or summary measures* | Table 3 |
| Main results | 16 | (a) Give unadjusted estimates and, if applicable, confounder-adjusted estimates and their precision (ego, 95% confidence interval). Make clear which confounders were adjusted for and why they were included | N/A |
|  |  | (b) Report category boundaries when continuous variables were categorized | N/A |
|  |  | (c) If relevant, consider translating estimates of relative risk into absolute risk for a meaningful time period | N/A |
| Other analyses | 17 | Report other analyses done—e.g. analyses of subgroups and interactions, and sensitivity analyses | None |
| Key results | 18 | Summarise key results with reference to study objectives | 9 |
| Limitations | 19 | Discuss limitations of the study, taking into account sources of potential bias or imprecision. Discuss both direction and magnitude of any potential bias | 10 |
| Interpretation | 20 | Give a cautious overall interpretation of results considering objectives, limitations, multiplicity of analyses, results from similar studies, and other relevant evidence | 10-11 |
| Generalisability | 21 | Discuss the generalisability (external validity) of the study results | 9-11 |
| **Other information** | |  |  |
| Funding | 22 | Give the source of funding and the role of the funders for the present study and, if applicable, for the original study on which the present article is based | 6 |

**Supplementary Material 2. Cultural characteristics of 38 countries**

| **Country** | **Power Distance** | **Individ-ualism** | **Success-Drivenness** | **Uncertainty Avoidance** | **Long-Term Orientation** | **Indul-gence** | **Health/**  **GDP** | **Gini** |
| --- | --- | --- | --- | --- | --- | --- | --- | --- |
| **Region 1: Central and Eastern Europe** | | | | | | | | |
| Armenia | - | - | - | - | 61 | - | 12.24 | 25.10 |
| Azerbaijan | - | - | - | - | 61 | 22 | 4.61 | - |
| Belarus | - | - | - | - | 81 | **15** | 6.41 | 24.40 |
| Bosnia and Herzegovina | - | - | - | - | 70 | 44 | 9.84 | 33.00 |
| Bulgaria | 70 | 30 | 40 | 85 | 69 | 16 | 8.52 | 40.50 |
| Croatia | 73 | 33 | 40 | 80 | 58 | 33 | 7.77 | 29.50 |
| Czech Republic | 57 | 58 | 57 | 74 | 70 | 29 | 9.24 | 26.20 |
| Estonia | 40 | 60 | 30 | 60 | 82 | 16 | 7.75 | 30.70 |
| Georgia | - | - | - | - | 38 | 32 | 7.60 | 34.50 |
| Hungary | 46 | **80** | **88** | 82 | 58 | 31 | 7.25 | 29.70 |
| Latvia | 44 | 70 | **9** | 63 | 69 | **13** | 7.45 | 35.70 |
| Lithuania | 42 | 60 | 19 | 65 | 82 | 16 | 7.54 | 36.00 |
| Moldova | - | - | - | - | 71 | 19 | 6.78 | 26.00 |
| Poland | 68 | 60 | 64 | 93 | 38 | 29 | 6.49 | 28.80 |
| Romania | **90** | 30 | 42 | 90 | 52 | 20 | 6.27 | 34.80 |
| Russia | **93** | 39 | 36 | 95 | 81 | 20 | 7.60 | 37.70 |
| Serbia | 86 | **25** | 43 | 92 | 52 | 28 | 8.73 | 34.50 |
| Slovakia | **100** | 52 | **100** | 51 | 77 | 28 | 7.23 | 23.20 |
| Ukraine | - | - | - | - | **86** | **14** | 7.62 | 26.60 |
| **Region 2: Northern and Western Europe** | | | | | | | | |
| Austria | **11** | 55 | **79** | 70 | 60 | 63 | 11.47 | 29.80 |
| Belgium | 65 | 75 | 54 | 94 | **82** | 57 | 11.06 | 26.00 |
| Denmark | **18** | 74 | 16 | **23** | **35** | **70** | 10.53 | 27.50 |
| Finland | 33 | 63 | 26 | 59 | 38 | 57 | 9.61 | 27.10 |
| France | 68 | 71 | 43 | 86 | 63 | 48 | 12.21 | 30.70 |
| Germany | 35 | 67 | 66 | 65 | **83** | 40 | 12.82 | 31.70 |
| Ireland | **28** | 70 | 68 | **35** | **24** | 65 | 7.10 | 29.20 |
| Netherlands | 38 | **80** | 14 | 53 | 67 | 68 | 11.14 | 29.20 |
| Norway | 31 | 69 | **8** | 50 | 35 | 55 | 11.42 | 27.70 |
| Slovenia | 71 | **27** | 19 | 88 | 49 | 48 | 7.23 | 23.20 |
| Sweden | 31 | 71 | **5** | **29** | 53 | **78** | 11.38 | 29.30 |
| Switzerland | 34 | 68 | 70 | 58 | 74 | 66 | 11.80 | 33.10 |
| United Kingdom | 35 | **89** | 66 | **35** | 51 | **69** | 11.98 | 32.80 |
| **Region 3: Southern Europe** | | | | | | | | |
| Greece | 60 | 35 | 57 | **100** | 45 | 50 | 9.51 | 33.60 |
| Italy | 50 | 76 | 70 | 75 | 61 | 30 | 9.63 | 35.20 |
| Malta | 56 | 59 | 47 | **96** | 47 | 66 | 10.84 | 31.40 |
| Portugal | 63 | **27** | 31 | **99** | **28** | 33 | 10.55 | 32.80 |
| Spain | 57 | 51 | 42 | 86 | 48 | 44 | 10.71 | 34.30 |
| Turkey | 66 | 37 | 45 | 85 | 46 | 49 | 4.62 | 41.90 |

**Top and bottom three scores are in bold.** “-“ = data unavailable.
